# Knowledge, attitude and experience of episiotomy use among obstetricians and midwives in Viet Nam

**DOI:** 10.1186/s12884-015-0531-2

**Published:** 2015-04-23

**Authors:** Anh T Trinh, Christine L Roberts, Amanda J Ampt

**Affiliations:** Hung Vuong Hospital, 128 Hong Bang Street, Ward 5, Ho Chi Minh City, Vietnam; Clinical and Population Perinatal Health Research, Kolling Institute, The University of Sydney at Royal North Shore Hospital, Building 52, Royal North Shore Hospital, St Leonards, NSW 2065 Australia

**Keywords:** Episiotomy, Knowledge, Attitudes, Experience, Survey, Viet Nam

## Abstract

**Background:**

Episiotomy remains a routine procedure at childbirth in many South-East Asian countries but the reasons for this are unknown. The aim of this study was to determine the knowledge of, attitudes towards and experience of episiotomy use among clinicians in Viet Nam.

**Methods:**

All obstetricians and midwives who provide delivery care at Hung Vuong Hospital were surveyed about their practice, knowledge and attitudes towards episiotomy use. Data were analysed using frequency tabulations and contingency table analysis.

**Results:**

148 (88%) clinicians completed the questionnaire. Fewer obstetricians (52.2%) than midwives (79.7%) thought the current episiotomy rate of 86% was about right (P < 0.01). Most obstetricians (82.6%) and midwives (98.7%) reported performing episiotomies on nulliparous women over 90% of the time. Among multipara, 24.6% of obstetricians reported performing episiotomy less than 60% of the time compared with only 3 (3.8%) midwives (P < 0.01). Aiming to reduce 3rd-4th degree perineal tears was the most commonly reported reason for performing an episiotomy by both obstetricians (76.8%) and midwives (82.3%), and lack of training in how to minimize tears and keep the perineum intact was the mostly commonly reported obstacle (obstetricians 56.5%, midwives 36.7% P = 0.02) to reducing the episiotomy rate.

**Conclusion:**

Although several factors that may impede or facilitate episiotomy practice change were identified by our survey, training and confidence in normal vaginal birth without episiotomy is a priority.

## Background

Episiotomy remains a common, or even routine, surgical procedure at childbirth in many South-East Asian countries [1-3]. For example, the episiotomy rate reported for Thailand in 2005 was 91% and for the Philippines was 64% compared with contemporaneous rates for Australia (17%) and the United states (25%) [3-5] High rates in South-East Asian countries persist despite randomised controlled trials which suggest that there are maternal benefits for using of selective episiotomy (when medically indicated) rather than routine use of the procedure [6]. Infant outcomes are similar for both approaches [6]. The reasons for ongoing use in South-East Asian countries are unclear, but lack of training, difference in culture and tradition, physiological differences between Asian and Caucasian women and fear of severe perineal injury have been speculated as reasons for the high rates [3,5,7]. With a view to informing practice changes that might reduce the episiotomy rate in Viet Nam, reliable and current information about clinician attitudes regarding episiotomy was needed. Therefore, the aim of this study was to determine the knowledge of, attitudes towards and experience of episiotomy use among obstetricians and midwives in a Vietnamese maternity hospital.

## Methods

The study was conducted in a maternity hospital in Ho Chi Minh City, Viet Nam between November 2012 and May 2013. Hung Vuong Hospital is one of the two biggest tertiary obstetrics hospitals in Ho Chi Minh City and on average cares for approximately 40,000 women and their newborn babies each year. Midwives are responsible for managing the uncomplicated pregnancies and normal vaginal births. Obstetricians manage all high risk and operative deliveries. In 2013, at Hung Vuong Hospital, the episiotomy rate among vaginal deliveries was 86%.

All 168 obstetricians and midwives who provide delivery care at Hung Vuong Hospital were eligible to complete a questionnaire in Vietnamese about their practice, knowledge and attitudes towards episiotomy use. Information collected on participant characteristics included profession (obstetrician or midwife), gender, and years of experience in maternity care (<5, 5–10, 11–30, >30 years). The practice questions included the frequency of episiotomy use among nulliparous and multiparous women, type of episiotomy used (midline/median, mediolateral [7–8 o’clock] or mediolateral [4–5 o’clock]) and the reasons for episiotomy use (including the main reason). Knowledge of the outcomes associated with routine episiotomy use was assessed using questions developed from a review of literature (including a Cochrane Systematic Review and observational studies), and included risk of postpartum haemorrhage, fetal distress, wound healing/complications, perineal pain, urinary incontinence and pelvic organ prolapse [5,6,8-13]. Although the Cochrane Review finds a policy of selective (compared with routine) episiotomy reduces the risk of severe perineal trauma (defined as third or fourth degree perineal laceration), there is debate about whether this policy is generalisable to South East Asian women who are not represented in any of the included randomised controlled trials [5,6]. Thus responses to a knowledge question about the association of routine episiotomy and severe perineal trauma could reflect either what the clinicians believe is true for the population they serve, or a knowledge of the Cochrane results, and hence interpretation may lack clarity. Consequently, we sought to address this issue in the reasons for use and attitude questions, rather than a knowledge question. Finally, discussion with clinical staff informed the questions about attitudes to episiotomy, which included an opinion (too high, too low, about right) on the current 86% episiotomy rate, appropriateness of a policy of routine episiotomy use for nulliparae and multiparae, and perceived barriers to reducing the hospital episiotomy rate. Questionnaire development included pilot testing on 20 obstetricians and midwives. Minimal changes were required to the survey following pilot testing (eg additional options were added to the reasons for episiotomy use), so it was decided that re-piloting was not necessary. The paper-based questionnaire took 3 to 5 minutes to complete.

Midwives and obstetricians who provided care for women at the time of delivery and had the opportunity to perform episiotomies were eligible to participate. Staff providing only antenatal care, early labour care, postnatal care or care in the caesarean section operating theatres (with no opportunity to perform episiotomies) were not eligible. Department heads identified the number of eligible staff and distributed information about the study and the paper-based questionnaires on behalf of the study investigators. The anonymous questionnaire included an introduction and an invitation to complete the questionnaire, and reassured potential participants that there were no right or wrong answers. Completed questionnaires were returned to a departmental in-tray and were collected by a study investigator (ATT). As no identifying information was collected, follow-up of non-responders was not possible. Completion and return of the questionnaire constituted consent to participate. The study was approved by the Institutional Review Board of Hung Vuong Hospital.

Survey data were analysed using frequency tabulations and contingency table analyses. A knowledge score (ranging from 0 to 6) was determined for each participant by assigning a point for each correct answer to the six knowledge questions (namely, that episiotomy increases the risk of postpartum hemorrhage (PPH), perineal pain and wound complications but not fetal distress, urinary incontinence or pelvic organ prolapse), and zero for incorrect or ‘don’t know’ responses. Analyses stratified by clinician type (obstetrician or midwife) were pre-specified, and differences in responses were assessed using the test of two proportions. Mean knowledge scores and standard deviations (SD) were calculated and compared among obstetricians and midwives using a two sample t test. Analyses were carried out using EpiInfo^TM^ 7 (Centers, for Disease Control and Prevention, Atlanta, GA, USA).

## Results

One hundred and forty eight (88%) clinicians completed the questionnaire including 69 (80%) of 86 obstetricians and 79 (96%) of 82 midwives. All the midwives were female, as were 75% of the obstetricians. There was no significant difference between obstetricians and midwives in their years of experience delivering maternity care with 60 (41%) <5 years experience, 50 (34%) having 5 to 10 years experience and 38 (26%) with over 10 years experience. No clinician had more than 30 years experience.

All (100%) respondents reported that they performed episiotomies and used the mediolateral (7–8 o’clock) approach. Ninety nine percent of midwives reported performing episiotomies on nulliparous women over 90% of the time, compared with 83% of obstetricians (Table 1). Similarly among multiparae, obstetricians performed episiotomies less frequently with 25% of obstetricians performing episiotomy less than 60% of the time compared with only 3 (3.8%) midwives (P < 0.01, Table 1).

**Table 1:** **Use episiotomy among obstetricians and midwives at Hung Vuong Hospital, 2012-13**

**Use of episiotomy**	**Obstetricians**	**Midwives**	**P-value**
	**N = 69**	**N = 79**	
	**n (%)**	**n (%)**	
*Among nulliparae*			
Always (99-100%)	28 (40.6)	37 (46.8)	0.44
Over 90% of the time	29 (42.0)	41 (51.9)	0.23
60% - 90% of the time	8 (11.6)	1 (1.3)	<0.01
<60% of the time	4 ( 5.8)	0 ( 0.0)	0.03
*Among multiparae*			
Over 90% of the time	19 (27.5)	22 (27.9)	0.97
60% - 90% of the time	33 (47.8)	54 (68.4)	0.01
<60% of the time	17 (24.6)	3 ( 3.8)	<0.01
*Reasons for performing episiotomy* ^*^			
Reduce 3^rd^ and 4^th^ degree perineal laceration	53 (76.8)	65 (82.3)	0.41
Operative delivery	53 (76.8)	64 (81.0)	0.53
Thick/swollen perineum	22 (31.9)	55 (69.6)	<0.01
Easy to do sutures	16 (23.2)	23 (29.1)	0.41
Shorten the 2^nd^ stage of labour	13 (18.8)	28 (35.4)	0.02
Afraid of fetal distress	8 (11.6)	19 (24.1)	0.05
Other	3 ( 4.4)	7 (8.9)	0.28

*more than one response possible.

Aiming to reduce 3^rd^-4^th^ degree perineal tears was the most commonly identified reason for performing an episiotomy by both obstetricians (76.8%) and midwives (82.8%) (Table 1), and this was also the *main* reason for performing episiotomies by both obstetricians (42.6%) and midwives (63.6%. P = 0.03). The second most frequent main reason for performing episiotomies reported by obstetricians was operative delivery (24.5%) but this was infrequently reported as a main reason (6.3%) by midwives who do not perform operative deliveries. Midwives were more likely than obstetricians to report a swollen perineum and need to shorten the 2^nd^ stage of labour as a reason for performing episiotomy. Other reasons for performing episiotomy included dystocia/large fetal size (n = 6) and former episiotomy scars (n = 2).

Overall knowledge scores ranged from 0 to 6 with a mean of 3 and did not differ significantly among obstetricians (3.3 ± 1.6) and midwives (3.5 ± 1.3, p = 0.5). Similarly, there were few differences in the responses by obstetricians and midwives to the individual knowledge questions (Table 2). The proportion of correct responses ranged from 30% (identified increased risk of PPH with routine episiotomy compared to women without episiotomy, obstetricians) to 67% (identified wound healing was not faster following episiotomy compared to a 2^nd^ degree tear, midwives). For some questions the rate of ‘don’t know’ was >20%.

**Table 2:** **Knowledge of the outcome as associated with routine episiotomy, Hung Vuong Hospital, 2012-13**

**Knowledge of episiotomy outcomes with routine use**	**Obstetricians**	**Midwives**	**P-value**
	**N = 69**	**N = 79**	
	**n (%)**	**n (%)**	
Prevalence of postpartum haemorrhage*			
Higher in women with episiotomy†	21 (30.4)	36 (45.6)	0.06
Lower in women with episiotomy	5 ( 7.3)	8 (10.1)	0.54
Equal	25 (36.2)	28 (35.4)	0.92
Do not know	18 (26.1)	7 ( 8.9)	<0.01
Prevalence of fetal distress*			
Higher in women with episiotomy	1 ( 1.5)	4 ( 5.1)	0.23
Lower in women with episiotomy	3 ( 4.4)	24 (30.4)	<0.01
Equal†	46 (66.7)	38 (48.1)	0.02
Do not know	19 (27.5)	13 (16.5)	0.10
*Faster wound healing?* ^‡^			
Yes	16 (23.2)	17 (21.5)	0.81
No^†^	41 (59.4)	53 (67.1)	0.33
Don’t know	12 (17.4)	9 (11.4)	0.30
*Less perineal pain?* ^‡^			
Yes†	16 (23.5)	26 (32.9)	0.19
No	39 (57.4)	48 (60.8)	0.60
Don’t know	13 (19.1)	5 ( 6.3)	0.02
Urinary incontinence*			
Yes	10 (14.5)	19 (24.1)	0.14
No†	43 (62.3)	52 (65.8)	0.66
Don’t know	16 (23.2)	8 (10.1)	0.03
Pelvic organ prolapsed*			
Yes	18 (26.1)	27 (34.2)	0.29
No†	40 (58.0)	50 (63.3)	0.51
Don’t know	11 (15.9)	2 ( 2.5)	<0.01

*compared to women without an episiotomy.

^†^response to knowledge question that was considered correct.

^‡^compared to women with a 2^nd^ degree tear.

About half of obstetricians (52.2%) thought an episiotomy rate of 86% was about right and the other half thought it was too high, whereas 79.7% of midwives thought it was about right (P < 0.01, Table 3). Almost all midwives (97.5%) thought routine episiotomy was an appropriate policy for nulliparae, while 71.0% of obstetricians thought it was (P < 0.01, Table 3). In contrast, few obstetricians or midwives considered routine episiotomy as appropriate for multiparous women, 8.7% and 12.7% respectively. The latter was the only outcome associated with experience; clinicians with ≥5 years experience with were less likely to consider routine episiotomy an appropriate policy for multiparae (5.7% vs 12.3%, P = 0.015).

**Table 3:** **Attitudes to episiotomy among obstetricians and midwives at Hung Vuong Hospital, 2012-13**

**Attitudes to episiotomy**	**Obstetricians**	**Midwives**	**P-value**
	**N = 69**	**N = 79**	
	**n (%)**	**n (%)**	
*Episiotomy rate (of 86%) is*			
Too low	0 ( 0.0)	1 ( 1.3)	0.35
About right	36 (52.2)	63 (79.7)	<0.01
Too high	33 (47.8)	14 (18.0)	<0.01
*Routine episiotomy is appropriate for nulliparae*	49 (71.0)	77 (97.5)	<0.01
*Routine episiotomy is appropriate for multiparae*	6 ( 8.7)	10 (12.7)	0.44
Other obstacles to reducing episiotomy rates?*			
Not trained to minimize tears/keep perineum intact	39 (56.5)	29 (36.7)	0.02
No time to wait for the perineum to stretch	34 (49.3)	21 (26.6)	<0.01
Hard to change traditional practice	24 (34.8)	19 (24.1)	0.15
Women expect an episiotomy	3 ( 4.4)	14 (17.7)	0.01
Other	10 (14.5)	18 (22.8)	0.20

*more than one response possible.

Sixty two (89.9%) obstetricians and 64 (81.0%) midwives identified obstacles to reducing the episiotomy rate, while 5 (7.3%) obstetricians and 13 (16.5%, P = 0.09) midwives stated there were no obstacles. The three most common obstacles reported were a lack of training in how to minimise tears and keep the perineum intact, work overload such that there was insufficient time to wait for the perineum to stretch, and the difficulty of changing traditional practices, with the first two reported more frequently by obstetricians (Table 3). Patient expectations was infrequently cited as an obstacle to reducing episiotomy rates (18% of midwives, 4% of obstetricians, Table 3) ‘Other’ obstacles reported included concern about the ability to minimise 3^rd^-4^th^ degree tears (n = 13) and managing a swollen perineum (n = 11). When asked to flag the most important obstacle to reducing episiotomy rates both obstetricians and midwives reported lack of training (36.4% versus 32%, P = 0.63).

## Discussion

This survey is the first of its kind published for Asian countries. We found obstetricians and midwives differ in their use of, and attitudes towards, episiotomy. Obstetricians have slightly less frequent use of episiotomy and are more likely to think the existing rate is too high. However, obstetricians and midwives have similar knowledge of the outcomes associated with episiotomy. Perhaps this should be expected as they are trained in the same university systems, although the teaching of obstetricians and midwives is separated.

Concern about 3^rd^-4^th^ degree tears was both the most commonly reported reason and the primary reason for episiotomy for both obstetricians and midwives, and lack of training in delivering women with an intact perineum was reported as a major obstacle to reducing episiotomy rates. The latter is not surprising as in Viet Nam, textbooks and practical training of accoucheurs in normal birth management currently advocate routine use of episiotomy. A recent Canadian study suggests that obstetric training impacts on attitudes as younger obstetricians were more likely (91%) to consider routine episiotomy did more harm than good compared with older obstetricians (79%) [14].

Although the trial evidence suggests that a policy of selective episiotomy does not increase the risk of 3^rd^-4^th^ degree tears, none of the trials included South-East Asian women and there remains uncertainty about the generalisability of the evidence among Vietnamese and other Asian women [5,6]. Asian ethnicity is a risk factor for severe perineal trauma in high income countries, and shorter perineal length has been speculated as the reason [15-17]. However, a study among Chinese women in Hong Kong reported a similar mean perineal length to that reported for other populations [1]. Both uncertainty about the applicability of the evidence among Asian women and lack of training will need to be addressed if practice is to change in Viet Nam. An assessment of perineal length, to help allay local concerns that Vietnamese women are different to the populations usually represented in research studies, is currently underway.

Anecdotal reports about clinicians’ fear of severe perineal trauma were confirmed in this study, even though the current 3^rd^–4^th^ degree tear rate in the hospital (based on internal audit) was incredibly low at 0.03% in 2012. This is in comparison with rates of 1-4% that are typically reported internationally, including among Vietnamese women who gave birth in Australia [5,15,18,19]. If 3^rd^-4^th^ degree tears are considered an indicator of poor quality of care [19-21], this may have resulted in under-reporting in medical records. Of greater concern is that severe perineal trauma goes unrecognised and unrepaired [22]. Postpartum follow-up in Viet Nam is highly variable (e.g. return to the clinic, maternity ward, an obstetrician’s private clinic, another hospital or local health centre, or no follow-up), and maternal urogenitary and faecal incontinence outcomes as indicators of severe perineal trauma are unknown. An independent assessment of perineal status in a cohort of women immediately post-delivery and a postpartum survey of maternal health (including documenting postpartum care, and urogenitary and bowel health) are planned to assess these issues.

Only 4% of obstetricians and 18% of midwives felt that women expected to have an episiotomy and as such, differences between professionals’ own views and what they believe are the views of their patients is not an obstacle to practice change. The high percentage of obstetricians and midwives who stated that they performed an episiotomy over 90% of the time for nulliparous women (83% and 99% respectively) gives an indication of the potential difficulty in instigating change, not only because of the high episiotomy rate for each individual clinician, but also because the majority of their peers do the same. In a secondary analysis of one of the episiotomy trials, Klein et al. demonstrated the difficulty of behaviour change among obstetricians with strong beliefs about episiotomy [23]. It is worth noting that in our study, while routine episiotomy for multiparae was considered appropriate by only 9% of obstetricians and 13% of midwives, 28% of both obstetricians and midwives reported that they would perform an episiotomy for this group over 90% of the time. With lower rates and different beliefs about appropriateness of episiotomy, clinicians may be more amenable to change for multiparous women.

Factors that may impede or facilitate behaviour change were also identified by our survey. With such a high rate of clinicians who state they have not been trained to minimise tears and keep the perineum intact (57% of obstetricians and 37% of midwives), we propose to develop and evaluate a local training program which will include dialogue with medical and midwifery training programs in universities. The training program will need address existing attitudes and the reasons behind these attitudes. Approximately half the obstetricians reported that they had no time to wait for the perineum to stretch as an obstacle to reducing the episiotomy rate, however less than one-fifth stated that one of the reasons they performed an episiotomy was to shorten the second stage of labour. This discrepancy may need to be explored in order to better understand workload, time restraints and capacity issues.

It remains unclear what rate of episiotomy in Vietnamese hospitals would give the greatest benefits for the least harm. Many high income countries report episiotomy rates below 20% [24]. However, it is noteworthy that implementation of an intensive national intervention in Norway that reduced the 3^rd^-4^th^ degree rate by 44% (from 4.1% to 2.3% of vaginal deliveries) was accompanied by a small increase in the episiotomy rate from 17.8% to 19.1% (2004–2010) [18]. Delivery unit clinical staff were involved in a multi-pronged education program that included techniques for conducting selective mediolateral episiotomies with emphasis given to the correct angle of incision, manual support of the perineum with good visualisation and good communication between the accoucheur and the labouring woman [25].

The strength of this study lies in the collection of standard information from both obstetricians and midwives reflecting current practice. We believe the high response rate reflects strong local interest in this topic and ensures the respondents were representative of the eligible population and the predominantly young, female maternity care workforce at Hung Vuong Hospital. While it is possible that some staff did not actually receive the questionnaire, we consider this unlikely. The number of eligible participants at the time of the survey was identified by department heads who also distributed questionnaires. Furthermore, we do not believe that our findings are subject to social desirability bias. A desired response would have been familiarity with evidence-based medicine, and the finding that most clinicians believe routine episiotomy is appropriate for nulliparous women is not consistent with best evidence. Although the study was limited to a single maternity hospital, the findings are likely to be generalisable to other maternity hospitals in Viet Nam as medical and midwifery training is university-based and not different across hospitals. With 90% of Vietnamese women birthing in a public or private health facility with a skilled birth attendant (obstetrician, nurse or midwife), our findings will have relevance for the majority of Vietnamese women giving birth [26].

## Conclusion

We have identified that the obstetricians and midwives in Viet Nam have certain beliefs about the reasons and consequences of performing an episiotomy that contradict current research evidence. Entrenched practices and attitudes indicate that changing episiotomy practice in Viet Nam will not be easy. However, we believe that patience and small incremental changes will be the best approach to achieving optimal outcomes for mothers and babies. This study is one step in a planned program of work that is attempting to facilitate practice change in Viet Nam.
